# Supplementary material for: Variation in Cause-Specific Mortality Rates in Italy during the First Wave of the COVID-19 Pandemic: A Study Based on Nationwide Data
Source: Int J Environ Res Public Health. 2022 Jan 12;19(2):805. doi: 10.3390/ijerph19020805 (PMC8776013; doi:10.3390/ijerph19020805)
Supplement: Supplementary file 1 [file ijerph-19-00805-s001.zip › ijerph-1480789-supplementary.pdf]

Supplementary material

Variation in Cause-Specific Mortality Rates in Italy during the First Wave of the COVID-19 Pandemic: A Study Based on Nationwide Data

Enrico Grande*[1], Ugo Fedeli[2], Marilena Pappagallo[1], Roberta Crialesi[1], Stefano Marchetti[1], Giada Minelli[3], Ivano Iavarone[4], Luisa Frova[1], Graziano Onder[5] and Francesco Grippo[1]

1. Italian National Institute of Statistics, Integrated system for health, social assistance and welfare, 00198 Rome, Italy
2. Epidemiological Department, Azienza Zero, Veneto Region, 35132 Padova, Italy
3. Statistical Service, Istituto Superiore di Sanità, 00161 Rome, Italy.
4. Department of Environment and Health, Istituto Superiore di Sanità, 00161 Rome, Italy
5. Department of Cardiovascular, Endocrine-metabolic Diseases and Ageing, Istituto Superiore di Sanità, 00161 Rome, Italy

Correspondence to:
Dr. Enrico Grande
Italian National Institute of Statistics
Viale Liegi 13, 00198 Rome, Italy
Email: grande@istat.it

Data completeness

Tables S1 and S2 show the number of deaths from the cause-of-death (CoD) register compared with the figures from the database of the demographic source, by geographical area and age class. Taking the latter as the reference, the percentage of deaths included in the CoD register is an estimate of its completeness. Nevertheless, a small difference between the two sources can be expected: the CoD register includes all deaths occurred in Italy, but not those of residents died abroad, which are instead included in the demographic source. The completeness of 2020 data is above 94% and there are only small differences by geographical area. Some differences in the completeness are observed by age groups.

Table S1. Comparison of the total number of deaths between CoD register and the demographic source database by geographical area.

Area	March-April 2020			March-April 2015-2019		
	CoD register	Demographic source (Daily mortality database)	CoD register/ demographic source	CoD register	Demographic source (daily mortality database)	CoD register/ demographic source
Lombardy	41,027	42,968	95.5	81,602	83,404	97.8
Other Northern regions	48,206	51,405	93.8	166,886	170,660	97.8
Rest of Italy	60,978	64,937	93.9	290,057	296,278	97.9
Italy	150,211	159,310	94.3	538,545	550,342	97.9

Table S2. Comparison of the total number of deaths between CoD register and the demographic source database by age.

Age group (years)	March-April 2020			March-April 2015-2019		
	CoD register	Demographic source (Daily mortality database)	CoD register/ demographic source	CoD register	Demographic source (daily mortality database)	CoD register/ demographic source
< 50	2,699	3,068	88.0	15,430	15,963	96.7
50-64	10,389	11,024	94.2	42,245	43,433	97.3
65-79	37,811	40,048	94.4	136,295	139,923	97.4
≥ 80	99,312	105,170	94.4	344,575	351,023	98.2

Estimation of the number of deaths by cause

The procedure consists in applying the proportion of deaths from specific causes in the CoD register to number of deaths from the demographic source stratified by geographical area, age, sex and month of death. The dataset with all deaths registered in Italy from the demographic source was downloaded by the Istat website, release of April 29th, 2021 (https://www.istat.it/it/archivio/240401).

Data in CoD and demographic source were stratified for the following variables:

- Province of residence
- Month of death
- Sex
- Age group, years (0-29, 30-49, 50-54, 55-59, 60-64, 65-69, 70-74, 75-79, 80-84, 85-89, 90-94, 95-99, 100 and more).

Deaths of the CoD register were further stratified by cause of death and for each cause the proportion of deaths within the stratum has been calculated. These proportions have then been multiplied by the number of deaths of the corresponding stratum in the demographic source.

$$_cP_{p,m,s,e} = \frac{_cd_{p,m,s,e}}{d_{p,m,s,e}}$$

Where d: number of deaths from CoD register, c: cause of death, p: province, m: month of death, s: sex, e: age, $_cP$: proportion of death for the cause c on the total number of deaths.

$$_c\widehat{D}_{p,m,s,e} = \ _cP_{p,m,s,e} \quad D_{p,m,s,e}.$$

Where D is the number of deaths in the demographic source and $_c\widehat{D}$, the estimated number of deaths for the cause c in each stratum.

When the number of deaths from the CoD register was lower than 50% of the demographic source, the proportion by cause used for the estimates was calculated at regional (larger territorial units, in Italy there are 20 regions) instead of at provincial level (in Italy there are 107 provinces).

In Table S3 the comparison between the number of deaths observed in the CoD register and the estimated number of deaths was reported.

Table S3. The number of deaths in the CoD register and the estimated number of deaths by cause from March 2020 to April 2020 compared with those in 2015–2019 (average).

Causes of death	March-April 2020				March-April 2015-2019 (average)			
	Number of deaths derived from CoD register		Estimated number of deaths		Number of deaths derived from CoD register		Estimated number of deaths	
	N	%	N	%	N	%	N	%
Covid-19	27,650	18.4	29,184	18.3				
Neoplasms	28,183	18.8	29,973	18.8	29,326	27.2	30,022	27.3
Diseases of the circulatory system	42,221	28.1	44,782	28.1	38,388	35.6	39,175	35.6
Diseases of the respiratory system	15,439	10.3	16,353	10.3	9,222	8.6	9,417	8.6
Symptoms, signs and ill-defined causes	4,498	3.0	4,766	3.0	3,329	3.1	3,399	3.1
Other causes	32,220	21.4	34,252	21.5	27,444	25.5	28,056	25.5
Total	150,211	100.0	159,310	100.0	107,709	100.0	110,068	100.0

Causes of death analysed

Table S4. Causes of death analyzed in the study and corresponding ICD10 codes.

**Cause of death**	**ICD10 codes**
COVID-19	U071-U072
Infectious and parasitic diseases (excluding Covid-19)	A00-B99
Neoplasms	C00-D48
Diabetes	E10-E14
Dementia and Alzheimer's disease	F01-F03, G30
Hypertensive diseases	I10-I15
Ischemic heart disease	I20-I25
Cerebrovascular diseases	I60-I69
Other diseases of the circulatory system	I00-I09, I26-I45, I47-I52, I70-I99
Influenza and pneumonia	J09-J18, J849
Chronic lower respiratory diseases	J40-J47
Other diseases of the respiratory system	J00-J06, J20-J39, J60-J848, J85-J99
Symptoms, signs and ill-defined diseases	R00-R99, I46
Transport accidents	V00-V99
Accidental falls	W00-W19
Suicide	X60-X84
Other external causes	W20-X59, X85-Y98
Other causes	D50-E07, E15-E90, F04-G26, G31-H95, K00-Q98
All causes excluding COVID-19	A00-Y98 excluding U071-U072
All causes	

Table S5. Age-standardized mortality rates from March 2020 to April 2020 by age and gender compared with those in 2015–2019 (values per 100,000 residents).

	**Age class (years)**							
	**<50**		**50-64**		**65-79**		**≥ 80**	
Cause of death	2020	2015-2019	2020	2015-2019	2020	2015-2019	2020	2015-2019
	(Males)							
COVID-19	1.3		25.9		156.5		537.1	
Neoplasms	2.8	2.9	36.3	40.0	157.2	170.5	436.2	450.6
Diabetes	0.1	0.1	3.1	2.5	18.4	14.3	95.9	67.8
Dementia and Alzheimer's disease		0.0	0.3	0.3	10.7	7.9	139.2	107.2
Hypertensive diseases	0.1	0.1	2.0	2.0	14.1	10.1	163.8	126.1
Ischemic heart disease	0.6	0.7	8.3	10.0	42.9	42.5	276.8	267.1
Cerebrovascular diseases	0.4	0.3	3.6	3.2	25.4	23.0	207.0	206.5
Other diseases of the circulatory system	0.6	0.6	5.0	4.8	26.3	27.2	243.2	238.5
Influenza and pneumonia	0.4	0.1	3.5	0.8	30.8	5.7	160.5	68.2
Chronic lower respiratory diseases	0.1	0.1	1.5	1.5	17.3	15.6	160.6	135.9
Other diseases of the respiratory system	0.3	0.1	1.3	1.1	8.1	7.8	62.8	55.9
Symptoms, signs and ill-defined diseases	0.9	0.9	3.5	3.2	8.6	6.9	82.8	62.1
Transport accidents	0.5	1.1	0.6	1.5	0.7	1.9	1.8	3.7
Accidental falls	0.1	0.1	0.4	0.5	2.8	2.0	19.2	14.4
Suicide	1.0	1.1	1.7	2.5	2.3	2.8	4.9	4.5
All causes excluding COVID-19	10.5	11.7	86.7	89.1	427.3	391.5	2375.9	2109.2
All causes	11.9	11.7	112.7	89.1	583.7	391.5	2913.0	2109.2
	(Females)							
COVID-19	0.5		6.7		53.7		302.9	
Neoplasms	3.3	3.2	28.1	30.6	91.1	93.1	224.5	228.9
Diabetes	0.1	0.1	1.3	1.0	10.3	8.5	77.6	58.6
Dementia and Alzheimer's disease	0.0	0.0	0.2	0.3	10.1	7.7	177.0	124.7
Hypertensive diseases	0.1	0.0	0.6	0.7	9.0	7.6	173.1	133.8
Ischemic heart disease	0.2	0.1	1.9	2.1	14.0	16.1	169.1	172.2
Cerebrovascular diseases	0.3	0.2	1.9	1.9	17.5	15.3	198.8	187.2
Other diseases of the circulatory system	0.3	0.3	1.5	2.1	15.7	16.3	205.0	196.2
Influenza and pneumonia	0.1	0.1	1.2	0.4	11.1	3.0	98.2	40.5
Chronic lower respiratory diseases	0.0	0.0	0.7	0.8	7.7	6.9	72.1	59.2
Other diseases of the respiratory system	0.1	0.1	0.7	0.5	4.6	3.8	42.1	34.3
Symptoms, signs and ill-defined diseases	0.3	0.3	1.3	1.0	4.5	3.8	82.7	60.2
Transport accidents	0.2	0.3	0.1	0.3	0.1	0.6	0.2	0.8
Accidental falls	0.0	0.0	0.2	0.1	1.0	0.8	10.8	8.5
Suicide	0.2	0.3	0.5	0.7	0.5	0.7	0.5	0.7
All causes excluding COVID-19	6.5	6.9	48.0	49.9	234.5	219.8	1782.8	1540.8
All causes	7.0	6.9	54.7	49.9	288.2	219.8	2085.7	1540.8

Table S6. Age standardized mortality rates (values per 100,000 residents) and 2020 to 2015-19 mortality rate ratios with 95% confidence intervals (CI) for all cause and all cause excluding COVID-19[a].

Sex	Cause of death	2020		2015-19			
		Age-standardized rates (with 95% CI)				Rate ratio 2020/2015-19	
Males	All causes	339.1	(336.7 - 341.6)	244.0	(243.0 - 245.0)	1.390	(1.378 - 1.401)
	All causes excluding COVID-19	268.2	(266.0 - 270.4)	244.0	(243.0 - 245.0)	1.099	(1.089 - 1.109)
Females	All causes	215.7	(214.2 - 217.2)	163.5	(162.9 - 164.1)	1.319	(1.309 - 1.330)
	All causes excluding COVID-19	183.0	(181.6 - 184.4)	163.5	(162.9 - 164.1)	1.119	(1.110 - 1.129)

[a] Mortality figures with 95% normal confidence intervals were computed using the SAS proc stdrate, assuming a Poisson distribution for the number of deaths.
